# *Chrysolina herbacea* Modulates Terpenoid Biosynthesis of *Mentha aquatica* L.

**DOI:** 10.1371/journal.pone.0017195

**Published:** 2011-03-09

**Authors:** Simon Atsbaha Zebelo, Cinzia M. Bertea, Simone Bossi, Andrea Occhipinti, Giorgio Gnavi, Massimo E. Maffei

**Affiliations:** Plant Physiology Unit, Department of Plant Biology, University of Turin, Innovation Centre, Turin, Italy; Instituto de Biología Molecular y Celular de Plantas, Spain

## Abstract

Interactions between herbivorous insects and plants storing terpenoids are poorly understood. This study describes the ability of *Chrysolina herbacea* to use volatiles emitted by undamaged *Mentha aquatica* plants as attractants and the plant's response to herbivory, which involves the production of deterrent molecules. Emitted plant volatiles were analyzed by GC-MS. The insect's response to plant volatiles was tested by Y-tube olfactometer bioassays. Total RNA was extracted from control plants, mechanically damaged leaves, and leaves damaged by herbivores. The terpenoid quantitative gene expressions (qPCR) were then assayed. Upon herbivory, *M. aquatica* synthesizes and emits (+)-menthofuran, which acts as a deterrent to *C. herbacea*. Herbivory was found to up-regulate the expression of genes involved in terpenoid biosynthesis. The increased emission of (+)-menthofuran was correlated with the upregulation of (+)-menthofuran synthase.

## Introduction

To deter herbivores, plants have evolved a broad range of defense mechanisms that can be generalized into two categories: pre-formed constitutive defenses and inducible defenses [1]. Constitutive defenses include the physical and chemical barriers that exist before insects attack, whereas induced defenses includes direct and indirect defenses. Direct defenses are plant traits that by themselves affect the susceptibility of host plants to insect attacks. Indirect defenses, on the other hand, include plant traits that by themselves do not affect the susceptibility of host plants, but can serve as attractants to natural enemies of attacking insects [2]–[7]. Insects may respond to plants by choosing different feeding sites, by altering their consumption rates or by induction of physiological/detoxification enzymes [8].

Approximately 90% of herbivorous insects have narrow host ranges, feeding on plants within a single taxonomic family, and many species are confined to a single host species [9]. The variability in fitness on different host plant species favors behavioral genotypes that restrict feeding to the most suitable hosts; on the other hand, maintaining mechanisms that nullify the disparate defensive adaptations of many different plant species is too costly for generalist herbivores [10]. Thus, the key to understanding why certain herbivores remain specialized lies in the observation that specialization involves both behavioral and physiological adaptation [8], [11], [12].

The defense strategy in aromatic plants like *Mentha aquatica* is a direct defense, through the constitutive production of terpenoids in specialized tissues known as the glandular trichomes [2]. These plants may have chemical barriers to potential herbivore colonists, and they appear to be accessible to relatively few insect lineages, which may be pre-adapted to chemically similar or related host plants [13]. As some insects become adapted to these metabolites, interactions between the two groups of organisms occasionally lead to highly specific relationships, as in the case of *M. aquatica* and the herbivore *Chrysolina herbacea*.

*M. aquatica*, or watermint, is a perennial plant belonging to the Lamiaceae. It produces leaf glandular trichomes secreting volatile organic compounds (VOCs) of varying chemical composition [14], [15]. The presence of the oxygenated monoterpenes (+)-pulegone and (+)-menthofuran contributes to the plant's toxicity. Both viridiflorol from the essential oil and (S)-naringenin from an ethanolic extract have been isolated by bioassay-guided fractionation with binding to the GABA-benzodiazepine site. Furthermore, *M. aquatica* contains psychoactive compounds that display both monoamine oxidase-inhibitory activity and mitochondrial respiration uncoupling [16]–[19]. At least 24 species of insect herbivores have been observed feeding on *M. aquatica* [20], [21], and among these, *C. herbacea*, also known as the mint beetle, is quite diffuse in mint fields. The feeding behavior of this beetle has been described recently [22]. Both larvae and adult beetles attack the leaves. Leaf beetles like *C. herbacea* are also known for their ability to import structurally distinct allelochemicals (reviewed by [23]). The ability to produce deterrents to natural enemies from plant-derived compounds is typical of some *Chrysolina* species [24]–[26]. Because herbivore feeding alters the aromatic profile of essential oil-producing plants like *M. aquatica*, the issue is both ecologically and economically relevant [27]–[29].

In this work we describe the chemical interaction between *M. aquatica* and *C. herbacea* by evaluating the ability of the herbivore to locate and recognize plant chemical cues and the capacity of the host plant to respond to herbivory by emitting deterrent molecules. To this end, plant VOC emissions we analyzed before and after herbivore feeding, and the ability of the emitted molecules to attract or deter *C. herbacea* was tested by bioassay.

## Results

### *C. herbacea* responds to *M. aquatica* VOC emission

Successful co-adaptation between plants and insects requires that plants produce specific compounds in response to herbivory, and that insects respond adequately to molecules emitted by plants. To look for possible relationships between *M. aquatica* VOC emissions and herbivore responses, the behavior of *C. herbacea* was first evaluated in Y-tube olfactometry tests.

**Figure 1:**
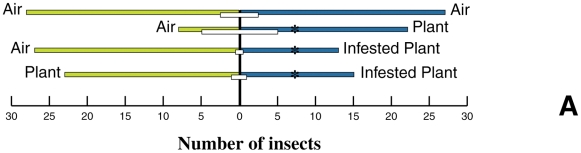
Response of *C. herbacea* in a Y-tube olfactometer when offered to *M. aquatica* cuttings of undamaged and *C. herbacea*-infested leaves versus pure GC-grade air. A χ^2^ test served to evaluate differences from a 50∶50 distribution over two olfactometer arms. Insects that did not reach the end of either olfactometer arm within 10 min (NC: no choice) are indicated by white bars. The asterisks indicate significant (P<0.05) differences.

*C. herbacea* was found to be attracted to undamaged plants, with respect to pure air. When the choice was between infested plants, undamaged plants or air, the insect was found to be deterred by infested plants (Fig. 1). Furthermore, the insects were found to lay eggs on undamaged plants and they produced larvae capable of surviving successive instars up to the adult phase (see Supporting Fig. S3). Preliminary studies demonstrated that the qualitative response of *M. aquatica* to larvae feeding was not significantly different from that of adult insects. For this reason, the study focused on adult insects.

### *M. aquatica* reacts to *C. herbacea* herbivory by emitting specific deterrent molecules

**Figure 2:**
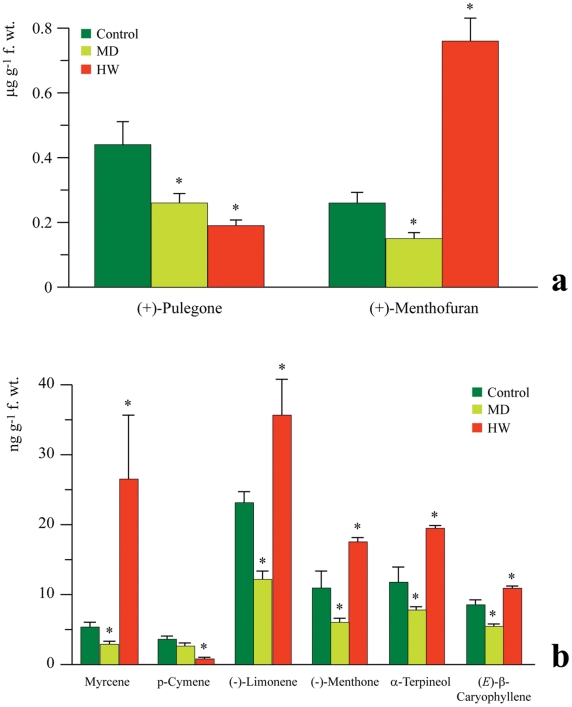
VOC emission by *M. aquatica* in undamaged plants (control), in response to mechanical damage caused by a pattern wheel (MD) and after herbivory by *C. herbacea* (HW). **a**, Content of the major components; **b**, content of the minor components. Bars indicate the standard error over the mean of at least three biological replicates. Asterisks indicate significant differences with respect to controls (P<0.05).

The reason why *C. herbacea* was differentially attracted to undamaged and infested plants was examined. VOCs emitted by untreated *M. aquatica* were analyzed by SPME, which revealed the presence of two major compounds, (+)-pulegone and (+)-menthofuran (Fig. 2a), along with other minor terpenoids including the monoterpenes (−)-limonene, (−)-menthone and α-terpineol, and the sesquiterpene, (*E*)-β-caryophyllene (Fig. 2b). However, it is important to note that the major compounds were emitted at much higher levels (0.13–0.75 µg g^−1^ f. wt) than the minor compounds in the range of 3–35 ng g^−1^ f. wt. The feeding activity of *C. herbacea* significantly changed the quantitative VOC composition of *M. aquatica* VOC emissions by dramatically increasing the content of (+)-menthofuran and decreasing the content of (+)-pulegone (Fig. 2a). The levels of all minor compounds, with particular reference to myrcene, were also elevated significantly upon herbivory; with the sole exception of *p*-cymene, which declined significantly (Fig. 2b). Since herbivory leads to the rupture of glandular trichomes, which are the main storage tissues of terpenoids in the Lamiaceae [2], [30], whether mechanical injury alone was able to increase the VOC emissions in *M. aquatica* was evaluated. Surprisingly, leaves damaged mechanically by a pattern wheel and having the same extent of herbivore damage had lower emissions of both major and minor compounds in comparison to control plants, particularly in comparison to herbivore wounded leaves (Figs. 2a and 2b).

### *C. herbacea* responds differentially to specific monoterpenes emitted by *M. aquatica*

**Figure 3:**
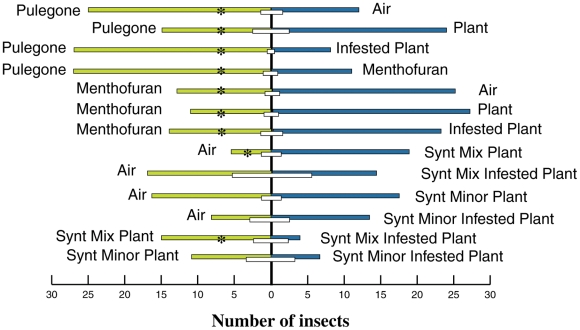
Response of *C. herbacea* in a Y-tube olfactometer when offered to either Sigma-grade (+)-pulegone, (+)-menthofuran, synthetic mixtures of compounds with the same content of typical intact plant emissions (Synt Mix Plant), synthetic mixtures of compounds with the same content of typical infested plant emissions (Synt Mix Infested Plant), synthetic mixtures of only minor compounds with the same content of typical intact plant emissions (Synt Minor Plant), synthetic mixtures of only minor compounds with the same content of typical infested plant emissions (Synt Minor Infested Plant) or GC-grade air. A χ^2^ test served toevaluate differences from a 50∶50 distribution over two olfactometer arms. Insects that did not reach the end of either olfactometer arm within 10 min (NC: no choice) are indicated by white bars. Asterisks indicate significant differences (P<0.05).

Following assessment of the chemical composition of the major VOCs released by undamaged and infested *M. aquatica*, the responses of *C. herbacea* to the main monoterpenes (+)-menthofuran and (+)-pulegone were tested. *C. herbacea* was significantly attracted to (+)-pulegone when the choice was limited to pure air, infested plants or (+)-menthofuran, whereas the insect preferred undamaged plants when offered with (+)-pulegone. On the contrary, (+)-menthofuran was always found to repel the insects, no matter which choice test was performed (Fig. 3). To evaluate whether the synthetic mixture composed of both major and minor compounds identified from leaf volatiles affected *C. herbacea* behavior, insect preference was determined by performing choice tests with several comparisons. *C. herbacea* was significantly attracted to the synthetic mixture typical of uninfested plants when compared to both air and a synthetic mixture typical of infested plants (Fig. 3). When only minor compounds of synthetic mixtures were compared to either air or other mixtures, no significant difference could be found (Fig. 3).

### Feeding of *C. herbacea* induces terpenoid gene expression in *M. aquatica*

**Figure 4:**
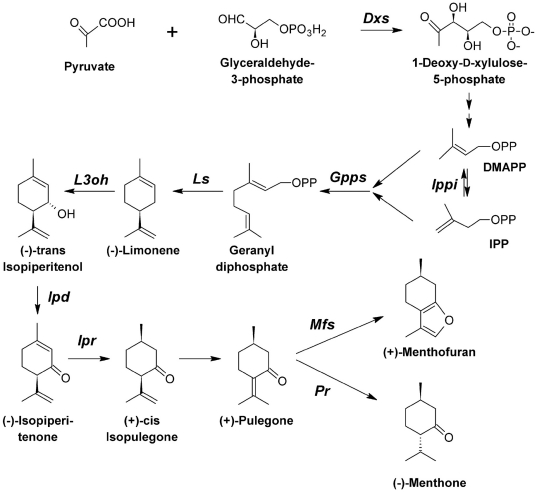
Schematic representation of the monoterpene biochemical pathway in *M. aquatica*. *Dxs*, 1-deoxy-D-xylulose-5-phosphate synthase; *Gpps* indicates geranyl diphosphate synthase; *Ls* indicates (−)-limonene synthase; *L3oh* indicates (−)-limonene-3-hydroxylase; *Ipd* indicates (−)-(3S,4R)-*trans*-isopiperitenol dehydrogenase; *Ipr* indicates (−)-isopiperitenone reductase; *Mfs* indicates (+)-menthofuran synthase; and *Pr* indicates (+)-pulegone reductase.

**Figure 5:**
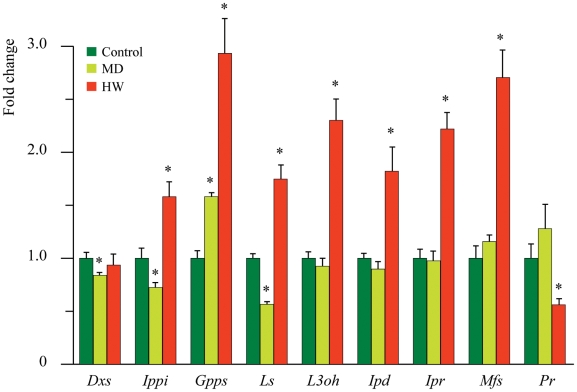
qPCR terpenoid gene expression of *M. aquatica* in mechanically damaged (MD) and herbivore damaged (HW) leaves, with respect to control leaves. Bars indicate the standard error over the mean of at least three biological replicates. Asterisks indicate significant differences with respect to controls (P<0.05).

Herbivory was found to affect the percentage of some monoterpenes emitted by *M. aquatica*. For this reason, gene expression involved in the biosynthetic pathway leading to the bioactive monoterpenes (+)-pulegone and (+)-menthofuran was evaluated. Previous studies have established the biochemical pathway that in mints that leads to the production of these two important monoterpenes (Fig. 4) [31]. We considered early genes such as *Dxs* and *Ippi*, which are involved in the formation and isomerization of the precursors isopentenyl diphosphate (IPP) and dimethylallyl diphosphate (DMAPP), respectively. *Dxp* showed no regulation by either herbivory or mechanical damage, whereas *Ippi* was down-regulated by mechanical damage and up-regulated by herbivory. *Gpps*, the gene coding for the enzyme that condenses one unit each of IPP and DMAPP into the monoterpene precursor geranyl pyrophosphate (GPP), was up-regulated almost 3-fold by herbivory and to a lesser extent by mechanical damage. *Ls*, coding for the enzyme conducting cyclisation of the universal precursor GPP to the parent olefin (−)-limonene, showed the same trend as *Ippi*, being up-regulated by herbivory and down-regulated by mechanical damage (Fig. 5). Several genes were always up-regulated by herbivory and showed no regulation after mechanical damage (Fig. 5). The genes included *L3oh*, which codes for the enzyme responsible for the NADPH- and O_2_-dependent hydroxylation of (−)-limonene to (−)-*trans*-isopiperitenol, *Ipd*, which codes the operationally soluble, NAD-dependent isopiperitenol dehydrogenase that catalyzes allylic oxidation to the α,β-unsaturated ketone (−)-isopiperitenone, *Ipr*, which codes the soluble enzyme (−)-isopiperitenone reductase that catalyzes the stereospecific, NADPH-dependent reduction of (−)-isopiperitenone to (+)-(1R,4R)-cis-isopulegone, and *Mfs*, which codes for menthofuran synthase, an enzyme responsible for the transformation of (+)-pulegone to (+)-menthofuran (Fig. 4). Isopulegone isomerase, which catalyzes double bond migration and causes the isopropenyl double bond of (+)-(1R,4R)-cis-isopulegone to yield (+)-pulegone, has not yet been isolated and was not assayed. Finally, *Pr*, which codes for pulegone reductase, the enzyme responsible for NADPH-dependent reduction of the conjugated double bond of the terpenone to yield (−)-menthone, was down-regulated by herbivory and showed no significant regulation upon mechanical damage (Figs. 4 and 5).

## Discussion

Over the past two decades it has been documented that plants produce blends of volatile compounds in vegetative tissues in response to damage and herbivore attack [4], [6], [32]–[38], suggesting that these substances act in plant defense [39]. Several lines of evidence indicate that VOCs released from vegetative tissues act as direct repellents against herbivores [40] and that the release of VOCs can result from the bursting of pre-existing structures in which volatiles are stored, such as glandular trichomes [41]. Plant VOCs can also attract natural enemies of attacking herbivores, such as parasitic wasps, flies, predatory mites or birds that can protect the signaling plant from further damage [7], [32], [37], [42], [43]

The results of this work show that *C. herbacea* is perfectly adapted to the blend of terpenoids emitted by undamaged *M. aquatica* and it uses this blend as a cue to locate plants. The fact that the insect lays eggs on undamaged plants is further evidence of such adaptation. In the case of the lepidopteran *Plutella xylostella*, the insect does not normally lay eggs on *Chrysanthemum morifolium*, because of the repellence of the monoterpene volatiles emitted from undamaged plants [44].

In the attracting blend of terpenoids produced by *M. aquatica*, (+)-pulegone was found to be the major compound in undamaged leaves and a potent attractant to *C. herbacea* in olfactometer bioassays. Volatiles emitted from plants can stimulate the behavioral or antennal responses of herbivores [45].

As a response to herbivore feeding, *M. aquatica* activates genes for terpenoid biosynthesis, diverting most of the terpene production toward the synthesis of (+)-menthofuran, which was found to repel *C. herbacea* in bioassay tests. Over the past decade, evidence that vegetative volatile compounds function to directly repel herbivores has begun to accumulate [39], [46]. However, the minor compounds emitted by uninfested and infested leaves were not effective in attracting or deterring *C. herbacea*.

An open question is why *M. aquatica* produces a herbivore attractant. One possible explanation is that the emission of (+)-pulegone is exploited by plants because of the antimicrobial [47], [48], nematocidal [49], acaricidal [50], antifeedant [51] and mitochondrial respiration uncoupling [19] properties of this compound. Preliminary studies have identified a *C. herbacea* egg parasite and studies on its behavior are under way.

This work suggests that constitutive plant defense can be modulated by interactions with herbivorous insects. The latter can trigger plant terpenoid gene expression and synthesis in a way that simple mechanical damage cannot. Usually, mechanical damage to plant foliage elevates VOC release in case of artificial damage carried out by researchers [40]; however, mechanically damaged *M. aquatica* does not exhibit emissions as intense or with the same compositional pattern as after herbivory.

Most genes directly involved in the biosynthesis of *p*-menthane monoterpenes in mints are transcriptionally regulated in a coordinated fashion [52] and it seems likely that the expression of these genes is controlled by a common transcription factor [53]. Herbivory had no effect on the expression of *M. aquatica Dxs*, a gene involved in the early steps of terpenoid biosynthesis for the mevalonate-independent (MEP)-pathway gene, the product of which is considered to catalyze one of the rate-limiting steps of this pathway [54]. On the contrary, herbivory up-regulated almost all other genes involved in the pathway. Over-expression of *Gpps*, *Ls* and *L3oh* would be expected to increase production of both GPP and other key monoterpenes [53]. The synthesis of (−)-limonene, providing the first committed intermediate of the pathway, is a possible rate limiting step of monoterpene production in mints [31]. Thus, the over-expression of both *Gpps* and *Ls* justifies the increase in the precursor (−)-limonene and the end-products of the pathway (particularly (+)-menthofuran). The up-regulation of *Mfs* parallels the increase in (+)-menthofuran; the latter was found to be a competitive inhibitor of Pr [55]. In transgenic lines with increased expression of *Mfs* and more (+)-menthofuran in the essential oil, (+)-pulegone amounts were greater than in controls. This finding led to the hypothesis that the metabolic fate of (+)-pulegone is controlled by (+)-menthofuran-mediated transcriptional down-regulation of Pr levels [56]. In *M. aquatica*, herbivory down-regulated the expression of *Pr* and up-regulated *Ipr*; however, the amounts of (+)-pulegone were never significantly different from those in the controls. This might indicate the presence of other factors that may exert post-translational control over the enzyme activity of Mfs or the regulation of the isopulegone isomerase, which has not yet been isolated.

In general, following herbivore attack, plants release green leaf volatiles (GLV), six-carbon aldehydes, alcohols, and esters, that are considered typical wound signals [4], [32], [57]–[59]. By contrast, significant amounts of green-leaf volatiles were not emitted by *M. aquatica* during herbivory. This may be related the high amount of terpenoids produced in the glandular trichomes; by comparison plants that do not accumulate these compounds in secretory tissues (e.g., *Arabidopsis* or Lima bean) usually released smaller amounts of terpenoids [36], [60]. In some cases it has been suggested that oxidative damage of membranes is one of the primary factors inducing GLV emissions [58]. The function of VOCs in alleviating oxidative stresses has been shown to be related to the high reactivity of certain monoterpenes [40], suggesting that the release of VOCs upon herbivory in *M. aquatica* might have other functions in addition to deterrence.

The proportions of emitted monoterpenes (VOCs) may differ from those in the plant oil of glandular trichomes with regard to chemical composition [27], [61]. Nonetheless, molecular data support the hypothesis that terpenoid biosynthesis is modulated by *C. herbacea* herbivory.

**Figure 6:**
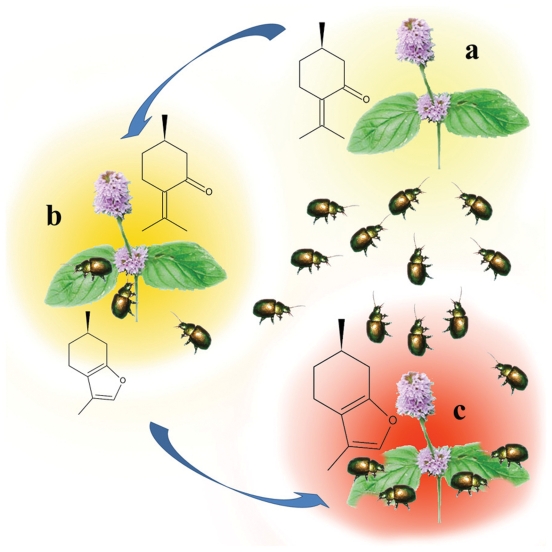
The behavior of *C. herbacea* and *M. aquatica* before and during herbivore feeding. **a**, Undamaged plants emit (+)-pulegone, which acts as an attractant for *C. herbacea*. **b**, Feeding activity induces gene expression and increases content of the deterrent compound (+)-menthofuran, along with the emission of the attractant compound (+)-pulegone; as a result, fewer insects are attracted to the plants. **c**, Intense feeding induces a reduction in (+)-pulegone content and a dramatic increase in the repellent compound (+)-menthofuran; *C. herbacea* avoids over-fed plants and moves towards undamaged plants.

In conclusion, *C. herbacea* attacks undamaged *M. aquatica*, but it avoids herbivore-infested *M. aquatica*. Upon herbivory, *M. aquatica* produces repellent compounds, thus reducing the damage from further insect attacks (Fig. 6, see also Supporting Fig. S4).

## Materials and Methods

### Plant material and growth conditions

Stolons of *Mentha aquatica* L. were collected from wild populations growing in Cambiano (Turin province, Italy, alt 240 m a.s.l.) and San Secondo di Pinerolo (Turin province, Italy, alt 413 m a.s.l.). Stolons were surface sterilized with 70% ethanol (Sigma-Aldrich, St. Louis, MO, USA) for 20 s and with sodium hypochlorite (1% v/v available chlorine) (Sigma-Aldrich) for 5 min. Stolons were then rinsed three times with sterile distilled water. Plants were grown in plastic pots with sterilized peat and vermiculite (V/V 4∶1) at 23°C and 60% humidity using daylight fluorescent tubes at 270 µE m^−2^ s^−1^ with a photophase of 16 h.

### Insect collection and rearing

Adults of *Chrysolina herbacea* (Duftschmid 1825) (Coleoptera, Chrysomelidae, Chrysomelinae) were collected by hand picking from infested *M. aquatica* fields. After collection, beetles were reared at 22°C in ventilated glass chambers and fed to *M. aquatica* cuttings. The beetles were starved for 24 h. prior the experiments.

### Collection of plant volatiles, gas chromatography and mass spectrometry

Experiments were conducted in 4 l glass desiccators by using non-flowering *M. aquatica* five-node cuttings, placed in 100 ml Erlenmeyer's flasks filled with 60 ml tap water and sealed with aluminium foil to prevent fall of insects into the water during experiments. Three cuttings per flask in a single desiccator where used. Plants were illuminated with fluorescent light bulbs generating about 50 µmol m^−2^ s^−1^ with a photophase of 16 h, the temperature inside desiccators was about 24°C and the relative humidity about 70%. Glass desiccators were connected to a GC-grade air generator (HPZA-3500-220, Parker Balston, Cleveland, OH, USA) through a cork plug with two openings allowing gases to go in and out. Air was pumped into the jars at a flow rate of 300 ml min^−1^. A clean Pasteur glass pipette was inserted in the outlet of the cork plug and VOCs were sampled with a Carboxen/Polydimethylsiloxane (CAR/PDMS) Supelco (Bellefonte, PA, USA) solid-phase micro-extraction (SPME) fibre (model 57334-U). Before use, SPME fibres were always conditioned at 250°C, according to manufacturer's instructions (see also Supporting Fig. S1 for more details).

Undamaged plants, leaves mechanically damaged with a pattern wheel and plants infested for 6 h were assayed for VOC emission. All experiments were standardized at 6 h, because the presence of eight herbivores for 6 h was found to cause about 30% of leaf damage.

SPME fibres, which were placed in various paths to adsorb VOCc for 6 h (see Supporting Figs. S1 and S2), were desorpted and VOCs were analyzed by gas-chromatography mass spectrometry (GC-MS 6890N-5973A, Agilent Technologies, Santa Clara, CA, US). The desorpted compounds were separated on a ZB-5MS Zebron (7HG-G010-11, Phenomenex, Torrance, CA, US) capillary column (stationary phase: polydimethylsiloxane - 5% diphenyl, 30 m length, 250 µm internal diameter, and 0.25 µm film thickness) with a temperature program of 60°C (kept for 5 min) followed by a temperature rise at a rate of 3°C min^−1^ to 270°C (kept for 5 min). Working conditions were: injector 250°C, transfer line to MSD 280°C, oven temperature: start 60°C, hold 5 min, programmed from 60°C to 270°C at 3°C min-1, hold 5 min; carrier gas was He under a constant flow of 1 ml min-1; in SPME desorption and subsequent analysis the fibre was exposed in the injection port during the entire GC run; the injector was maintained in splitless mode during the desorption phase; ionization energy: EI 70 eV; acquisition parameters: scanned m/z 50–250 amu. Separated compounds were identified by pure standard comparison, by comparison of their mass spectra and retention indexes (Kováts indexes) with those of reference substances and by comparison with the NIST mass spectral search software v2.0 using the libraries NIST 98 library and Adams [62] library. Different concentraitions of (+)-menthofuran, (+)-pulegone, myrcene, *p*-cymene, (−)-limonene, (−)-menthone, α-terpineol and (*E*)-β-caryophyllene were used to create a standard curve used as an external standard for SPME quantitative measurements. Reference compounds were mixed in relative proportions similar to those that were quantified in the plant samples.

### Total RNA extraction and quantitative Real Time-PCR (qPCR)

After each experiment, leaves were collected and immediately frozen in liquid nitrogen. One hundred mg of frozen control, herbivore damaged, and mechanically damaged leaves were ground in liquid nitrogen with mortar and pestle. Total RNA was isolated using Qiagen RNeasy Plant RNA kit and RNase-Free DNase set (Qiagen, Hilden, Germany). Sample quality and quantity was checked by using the RNA 6000 Nano kit and the Agilent 2100 Bioanalyzer (Agilent Technologies) according to manufacturer's instructions. Quantification of RNA was also confirmed spectrophotometrically by using a NanoDrop ND-1000 (Thermo Fisher Scientific, Waltham, MA, US).

First strand cDNA synthesis was accomplished with 2 µg of total RNA and random primers using the High-Capacity cDNA Reverse Transcription Kit (Applied Biosystems, Foster City, CA, US), according to the manufacturer's recommendations. Primers for real-time PCR were designed on *Mentha piperita* available sequences using the Primer 3 software [63]. qPCR was done on an Mx3000P Real-Time PCR System (Stratagene, La Jolla, CA, US). The reaction was performed with 25 µl of mixture consisting of 12.5 µl of 2× Maxima™ SYBR Green qPCR Master Mix (Fermentas International, Inc, Burlington, ON, Canada), 0.5 µl of cDNA and 100 nM primers (Integrated DNA Technologies, Coralville, IA, US).

Relative RNA levels were calibrated and normalized with the level of two housekeeping genes: actin and 18S ribosomal mRNA.

PCR conditions were determined by comparing threshold values in dilution series of the RT product, followed by non-template control for each primer pair. Relative expression levels of genes were calculated by using the Pfaffl method [64]. A suitable melt curve analysis was always performed.

PCR conditions were the following: *18S*: initial polymerase activation of 10 min at 95°C; and 40 cycles of 15 s at 95°C, 30 s at 58°C, and 30 s at 72°C; *Actin*: initial polymerase activation of 10 min at 95°C; and 40 cycles of 15 s at 95°C, 30 s at 57°C, and 30 s at 72°C; *Dxs*: initial polymerase activation of 10 min at 95°C; and 40 cycles of 15 s at 95°C, 30 s at 58°C, and 30 s at 72°C; *Ippi*: initial polymerase activation of 10 min at 95°C; and 40 cycles of 15 s at 95°C, 30 s at 57°C, and 30 s at 72°C; *Gpps*: initial polymerase activation of 10 min at 95°C; and 40 cycles of 15 s at 95°C, 30 s at 58°C, and 30 s at 72°C; *Ls*: initial polymerase activation of 10 min at 95°C; and 40 cycles of 15 s at 95°C, 30 s at 57°C, and 30 s at 72°C; *L3oh*: initial polymerase activation of 10 min at 95°C; and 40 cycles of 15 s at 95°C, 30 s at 57°C, and 30 s at 72°C; *Ipd*: initial polymerase activation of 10 min at 95°C; and 40 cycles of 15 s at 95°C, 30 s at 58°C, and 30 s at 72°C ; *Ipr*: initial polymerase activation of 10 min at 95°C; and 40 cycles of 15 s at 95°C, 30 s at 57°C, and 30 s at 72°C; *Pr*: initial polymerase activation of 10 min at 95°C; and 40 cycles of 15 s at 95°C, 30 s at 57°C, and 30 s at 72°C; *Mfs*: initial polymerase activation of 10 min at 95°C; and 40 cycles of 15 s at 95°C, 30 s at 58°C, and 30 s at 72°C.

Primers used for qPCR were the following: 18S, (NCBI GenBank accession no. NR_022795), forward primer 5′-ATGATAACTCGACGGATCGC-3′, reverse primer 5′-CTTGGATGTGGTAGCCGTTT -3′; actin, (NCBI GenBank accession no. AW255057), forward primer 5′-GCTCCAAGGGCTGTGTTCC-3′, reverse primer 5′- TCTTTCTGTCCCATGCCAAC-3′ [65]. *Dxs*, (NCBI GenBank accession no. AF019383), [66], forward primer 5′-CCACCAGGCTTACCCACACAA-3′, reverse primer 5′-GCCACCGCCATCCCTAAAC-3. *Ippi*, (NCBI GenBank accession no. AW255524), [65], forward primer 5′-CTCTTGGGGTGAGAAATGCT-3′ reverse primer 5′-CATCTGAGGGGGCTTTGTA-3. *Gpps*, (NCBI GenBank accession no. EU108696), [67], forward primer 5′-ATGATAAGCGGGCTGCATAG-3′ reverse primer 5′-CCGAAATTCCTCAGCTTCTG-3′. *Ls*, (NCBI GenBank accession no. AW255536), [65], forward primer 5′-CGGTGGTGGAGAAATACTGGGTTT-3′, reverse primer 5′-CCGTAATCAGAGCGTGACTTTGC-3′. *L3oh*, (NCBI GenBank accession no. AF124817), [68], forward primer 5′-CCCCATCACCACCAACTCCA-3′, reverse primer 5′-GCTCCGCCAGCACCCATAG-3′; *Ipd*, (NCBI GenBank accession no. AY641428), [69], forward primer 5′-GAGCTTCTATGGGCAGGTCA-3′, reverse primer 5′- GGCCACGAATGGTAAACACT-3′. *Ipr*, (NCBI GenBank accession no. AY300162), [70], forward primer 5′-AGCCAATGGAGAAATGATCG-3′, reverse primer 5′- GAGAGGAATGAGGGCTTGTG-3′. *Pr*, (NCBI GenBank accession no. AAQ75423), [70], forward primer 5′-ACAGCCTGAAGCAGCCTGAA-3′, reverse primer 5′-CGGCAGAACCATCTCAAGGA-3′. *Mfs*, (NCBI GenBank accession no. AF346833), [71], forward primer 5′-GCAGAACGAGGTGCGAGAAG -3′, reverse primer 5′-TGCGAAAGGTGGATGTAGGC-3′. The length of PCR products was from 98 to 200 bp.

### Bioassays

Experiments were conducted in a glass Y-tube olfactometer connected to the glass jars. GC-grade air was pumped into the jars at a flow rate of 300 ml min^−1^. The Y-tube was housed in a blackened box with a diffused fluorescent lamp giving a constant light directly above the centre point of the Y-tube. Thirty *C. herbacea* were monitored for up to 7 min by recording “choice” or “no-choice” events. All glassware was carefully washed to remove any contaminating substances and air-dried at 120°C for 4 h to remove volatile compounds. Test temperature was maintained at about 24°C and 70% humidity. (+)-Menthofuran, (−)-pulegone, myrcene, *p*-cymene, (−)-limonene, (−)-menthone, α-terpineol and (*E*)-β-caryophyllene pure standards were purchased from Sigma-Aldrich and were used in chemical tests (see also Supporting Fig. S2). The concentration of pure standards in Y-tube tests was used at the same concentrations as found in leaf volatile emissions.

### Statistical analysis

Initial and final choice data were analyzed using a Chi-squared test. Yates' correction was applied to adjust for the data with only one degree of freedom. Data were also statistically processed using a log-likelihood test (G-test) and by ANOVA by using the statistical program SPSS (version 16.0,SPSS Inc., Chicago, IL, USA). For genomic and chemical analyses, the overall data sets are expressed as mean values of at least three biological replicates each one repeated three times (technical replicates). Metric bars indicate SD. Significance of differences observed in data sets was tested by ANOVA using the SYSTAT 10 software.

## Supporting Information

Figure S1 Left panel, glass desiccators containing the plants are connected to the GC-grade air generator. The cork plug has two holes: one for allowing the GC-grade air to enter the jar and the other hosts a glass Pasteur pipette. In the right figure the arrow indicates the SPME fibre that adsorbs the VOCs exiting from the jar.
(PDF)

Figure S2 Left panel, a glass Y-tube olfactometer is connected to the jars where a flux of GC-grade air blows the VOCs produced by undamaged and infested leaves. Arrows indicate the presence of the SPME fibre which is located just before the olfactometer arms, into the air path. Right upper panel shows *C. herbacea* making a choice. The lower right panel shows the flow-meter used to standardize the air flow blowing from the jars and the timer used during the choice tests.
(PDF)

Figure S3 *C. herbacea* was found to lay eggs on undamaged *M. aquatica* plants. The left panel shows clutches of eggs laid on a young *M. aquatica* leaf. The right panel shows young *C. herbacea* larvae feeding on *M. aquatica* leaves.
(PDF)

Figure S4 *M. aquatica* plants observed in the wild. The upper left panel shows an undamaged *M. aquatica* in full bloom. The upper right panel shows male and female *C. herbacea* mating on partially damaged *M. aquatica* leaves. The two lower panels show the typical level of damage inferred by *C. herbacea* on wild *M. aquatica* leaves.
(PDF)
