# Supplementary material for: Chrysolina herbacea Modulates Terpenoid Biosynthesis of Mentha aquatica L
Source: PLoS One. 2011 Mar 9;6(3):e17195. doi: 10.1371/journal.pone.0017195 (PMC3052309; doi:10.1371/journal.pone.0017195)
Supplement: Figure S1 — Left panel, glass desiccators containing the plants are connected to the GC-grade air generator. The cork plug has two holes: one for allowing the GC-grade air to enter the jar and the other hosts a glass Pasteur pipette. In the right figure the arrow indicates the SPME fibre that adsorbs the VOCs exiting from the jar. (PDF) [file pone.0017195.s001.pdf]

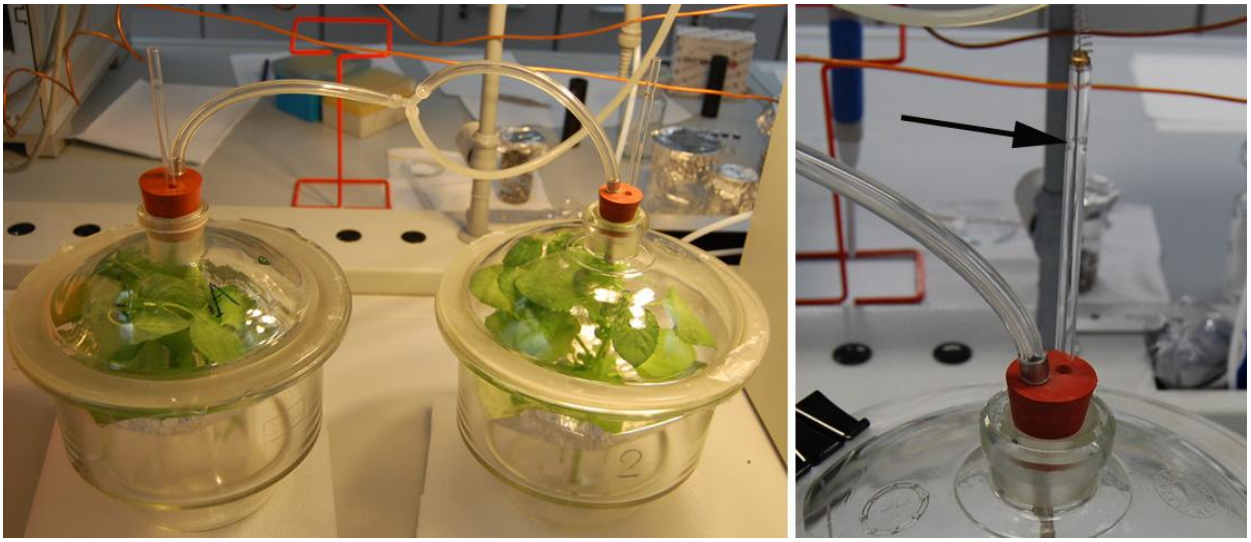

Figure S1. Left panel, glass desiccators containing the plants are connected to the GC-grade air generator. The cork plug has two holes: one for allowing the GC-grade air to enter the jar and the other hosts a glass Pasteur pipette. In the right figure the arrow indicates the SPME fibre that adsorbs the VOCs exiting from the jar.
