# Supplementary material for: Chrysolina herbacea Modulates Terpenoid Biosynthesis of Mentha aquatica L
Source: PLoS One. 2011 Mar 9;6(3):e17195. doi: 10.1371/journal.pone.0017195 (PMC3052309; doi:10.1371/journal.pone.0017195)
Supplement: Figure S2 — Left panel, a glass Y-tube olfactometer is connected to the jars where a flux of GC-grade air blows the VOCs produced by undamaged and infested leaves. Arrows indicate the presence of the SPME fibre which is located just before the olfactometer arms, into the air path. Right upper panel shows C. herbacea making a choice. The lower right panel shows the flow-meter used to standardize the air flow blowing from the jars and the timer used during the choice tests. (PDF) [file pone.0017195.s002.pdf]

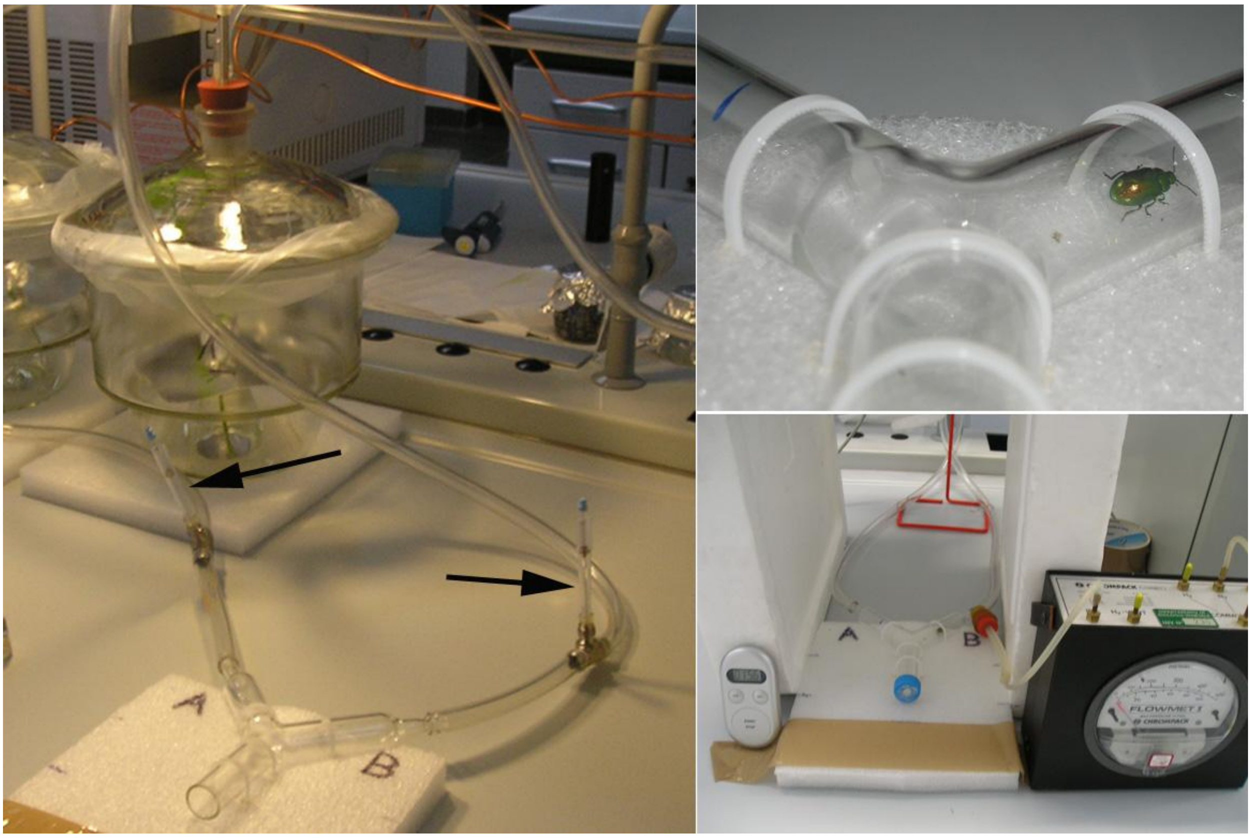

Figure S2. Left panel, a glass Y-tube olfactometer is connected to the jars where a flux of GC-grade air blows the VOCs produced by undamaged and infested leaves. Arrows indicate the presence of the SPME fibre which is located just before the olfactometer arms, into the air path. Right upper panel shows *C. herbacea* making a choice. The lower right panel shows the flow-meter used to standardize the air flow blowing from the jars and the timer used during the choice tests.
