# Supplementary material for: Chrysolina herbacea Modulates Terpenoid Biosynthesis of Mentha aquatica L
Source: PLoS One. 2011 Mar 9;6(3):e17195. doi: 10.1371/journal.pone.0017195 (PMC3052309; doi:10.1371/journal.pone.0017195)
Supplement: Figure S3 — C. herbacea was found to lay eggs on undamaged M. aquatica plants. The left panel shows clutches of eggs laid on a young M. aquatica leaf. The right panel shows young C. herbacea larvae feeding on M. aquatica leaves. (PDF) [file pone.0017195.s003.pdf]

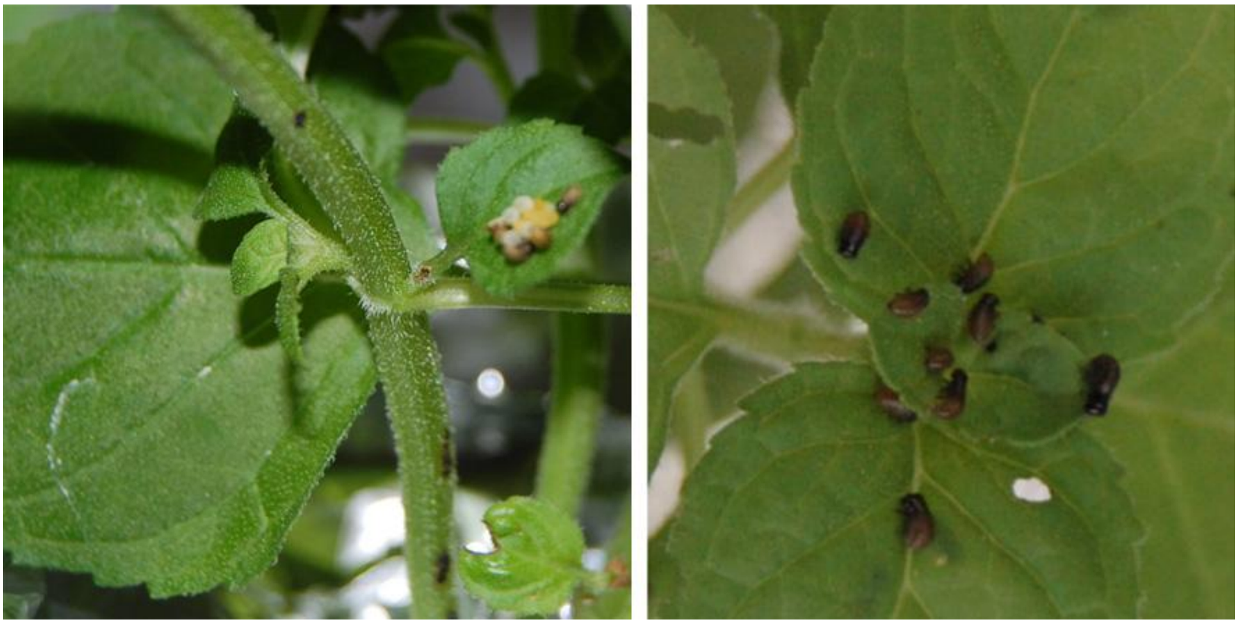

Figure S3. *C. herbacea* was found to lay eggs on undamaged *M. aquatica* plants. The left panel shows clutches of eggs laid on a young *M. aquatica* leaf. The right panel shows young *C. herbacea* larvae feeding on *M. aquatica* leaves.
