# Supplementary material for: Chrysolina herbacea Modulates Terpenoid Biosynthesis of Mentha aquatica L
Source: PLoS One. 2011 Mar 9;6(3):e17195. doi: 10.1371/journal.pone.0017195 (PMC3052309; doi:10.1371/journal.pone.0017195)
Supplement: Figure S4 — M. aquatica plants observed in the wild. The upper left panel shows an undamaged M. aquatica in full bloom. The upper right panel shows male and female C. herbacea mating on partially damaged M. aquatica leaves. The two lower panels show the typical level of damage inferred by C. herbacea on wild M. aquatica leaves. (PDF) [file pone.0017195.s004.pdf]

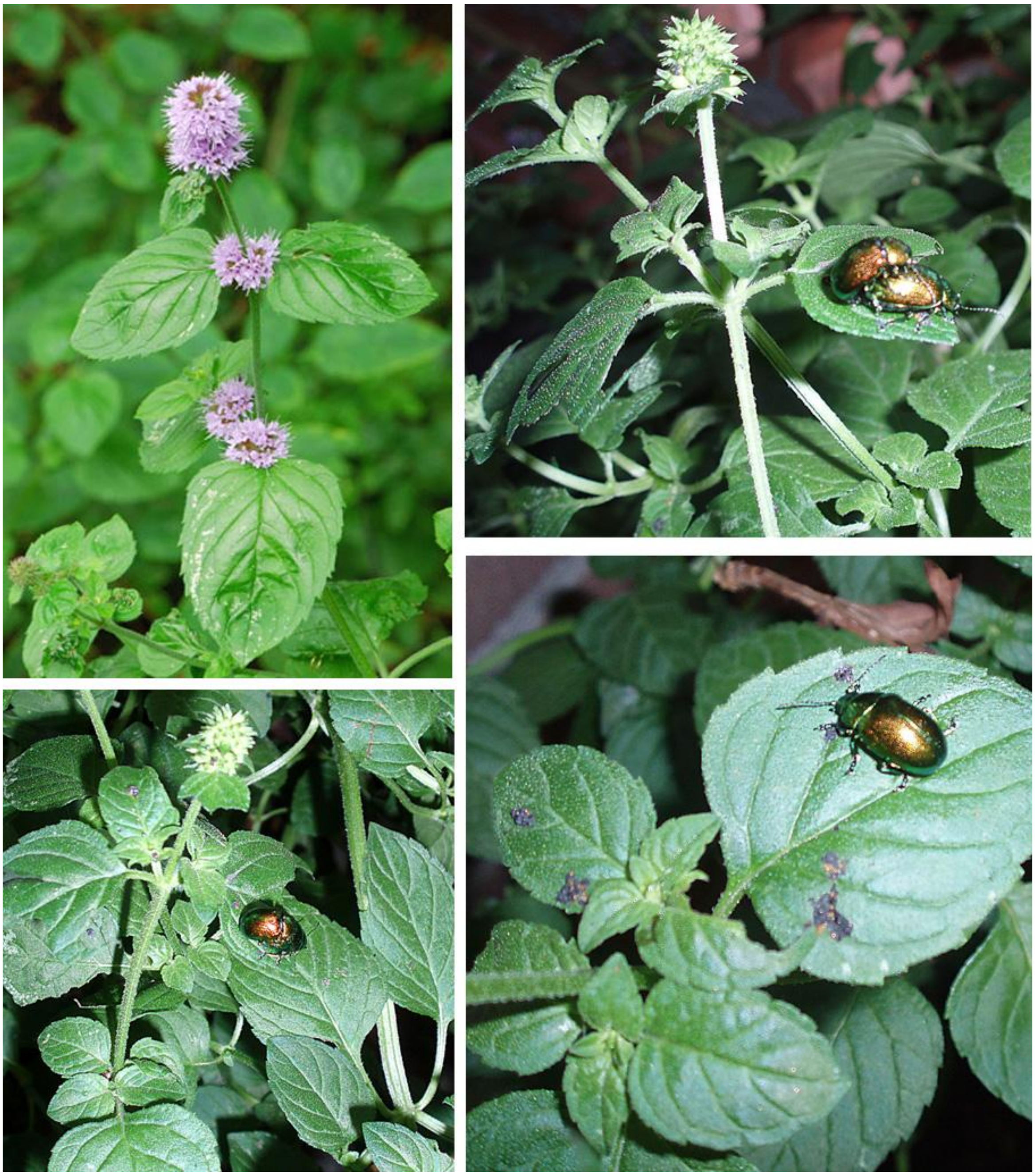

Figure S4. *M. aquatica* plants observed in the wild. The upper left panel shows an undamaged *M. aquatica* in full bloom. The upper right panel shows male and female *C. herbacea* mating on partially damaged *M. aquatica* leaves. The two lower panels show the typical level of damage inferred by *C. herbacea* on wild *M. aquatica* leaves.
